# Melatonin reduces migratory restlessness in *Sylvia* warblers during autumnal migration

**DOI:** 10.1186/1742-9994-10-79

**Published:** 2013-12-26

**Authors:** Leonida Fusani, Francesca Coccon, Alfonso Rojas Mora, Wolfgang Goymann

**Affiliations:** 1 Department of Life Sciences and Biotechnology, University of Ferrara, Ferrara, Italy; 2 Department of Environmental Science, Informatics and Statistics, Ca’ Foscari University of Venice, Venice, Italy; 3 Evolutionary Ecophysiology, Institute of Biology, University of Neuchatel, Neuchatel, Switzerland; 4 Abteilung für Verhaltensneurobiologie, Max-Planck-Institut für Ornithologie, Eberhard-Gwinner-Str. 6a, D-82319 Seewiesen, Germany

**Keywords:** Bird migration, Melatonin, Migratory restlessness, *Zugunruhe*, Nocturnal migration, Blackcap, Garden warbler, Songbird, Passeriformes, Avian

## Abstract

**Introduction:**

A remarkable aspect of bird migration is its nocturnality, particularly common in Passeriformes. The switch in activity from purely diurnal to also nocturnal is evident even in caged birds that during migratory periods develop an intense nocturnal restlessness, termed *Zugunruhe*. The mechanisms that control this major change in activity are mostly unknown. Previous work with *Sylvia* warblers suggested an involvement of melatonin, a hormone associated with day-night cycles in most vertebrates. In a recent study we found no effects of melatonin administration on *Zugunruhe* during spring migration. However, previous studies indicated that the response to melatonin manipulation could differ between spring and autumn migration, which are in fact separate life history stages. Here we tested whether a non-invasive treatment with melatonin can alter *Zugunruhe* in wild garden warblers *S. borin* and blackcaps *S. atricapilla* subject to temporary captivity at an autumnal stopover site. Food availability in the cage (yes/no) was added as a second factor because previous work showed that it enhanced *Zugunruhe*.

**Results:**

The melatonin treatment significantly decreased the amount of *Zugunruhe*, while the availability of food only tended to increase the amount of *Zugunruhe*. Fuel deposits also had a strong effect on the amount of nocturnal activity: lean birds with a fat score of 1 showed significantly less *Zugunruhe* than fatter birds. The change in body mass during the time spent in the recording cage depended on food availability, but not on any of the other factors.

**Conclusions:**

This study shows that the migratory programme of two *Sylvia* warblers can be manipulated by administration of exogenous melatonin and confirms that this hormone is involved in the control of migratory behaviour. To our knowledge, this is one of the first demonstrations that the autumn migratory programme can be altered by hormonal manipulation in migrating birds. The comparison with a similar study carried out with the same modalities during spring migration suggests that there are seasonal differences in the sensitivity of the migratory programme to hormonal factors. In birds breeding in the northern hemisphere, the importance of a timely arrival to the breeding sites could explain why the control of the migratory programme is more rigid in spring.

## Introduction

Nocturnal migration is common in many passerine species that are day-active during all other stages of their annual cycle. The adaptive aspects of migrating at night are debated, and may include a combination of factors including avoidance of overheating, thermal condition of the atmosphere, reduced predation risk, use of orientation cues at sunset or during the night, and optimization of foraging strategies [1,2]. In captive birds, the onset of migration is indicated by the periodic expression of intense restlessness at night. Although some non-migratory species or populations may show nocturnal restlessness, only birds that migrate over hundreds or thousands of kilometers show the robust locomotor activity that may persist for weeks and that has been traditionally called *Zugunruhe*[3,4].

Melatonin is a hormone secreted by the pineal gland (or epiphysis), and in most vertebrates is implicated in transducing the photic signal [5]. In birds, melatonin and the pineal gland are major players in the regulations of circadian rhythms [6]. Pioneer studies of Eberhard Gwinner and his colleagues had shown that in *Sylvia* warblers the amplitude of nocturnal melatonin secretion is reduced when birds show *Zugunruhe* compared to other times of the year [7], (reviewed by [8]). In addition, the correlation between nocturnal activity and reduced melatonin levels was present in migratory blackcaps *Sylvia atricapilla,* but not in a non-migratory population of this species which showed some nocturnal activity [4].

We hypothesized that melatonin is involved in the switch between the nocturnal activity typical for migration and the diurnal pattern of activity shown during all other stages of their annual cycle (such as breeding, moulting and wintering) [8,9]. Following this hypothesis, we made two predictions that could be tested empirically: First, if the birds were induced to interrupt *Zugunruhe* during migration, one should observe an increase of circulating melatonin at the same time. Second, treatment with exogenous melatonin should reduce the intensity of *Zugunruhe*.

In a first study, we tested the first prediction using a food deprivation protocol that had been developed previously to cause an interruption of *Zugunruhe*[10,11]. In this protocol, birds with substantial amounts of subcutaneous fat and that show robust *Zugunruhe* at night (i.e. birds that are in full migratory condition) are food-deprived for two consecutive days, simulating a long non-stop flight over ecological barriers such as deserts or seas. On the third day, food is reintroduced. As a consequence, most birds reduce or completely suppress *Zugunruhe* in the following night. The interpretation of this phenomenon (also called ‘Biebach effect’) is that birds react as if they were temporarily interrupting their migration to refuel upon reaching a suitable stopover site [10-13]. When we applied this protocol to blackcaps, we found that plasma levels of melatonin increased in birds that reduced their *Zugunruhe*[14]. Thus, the study confirmed the first prediction derived from the hypothesis that melatonin was involved in the expression of *Zugunruhe*.

The second prediction was recently tested in wild garden warblers *Sylvia borin* during spring migration [15]. We caught garden warblers on Ponza, an island in the Mediterranean Sea off the western coast of Italy that receives large numbers of migrants that crossed the sea coming from northern Africa. The birds were hosted overnight in activity registration cages, and just before dark, half of the birds were treated with a melatonin cream that had been previously validated to provide transient elevation of melatonin [16]. Despite the very large sample size (N = 196) for a hormone manipulation experiment we found no effects of melatonin on *Zugunruhe*[15]. Therefore, the second prediction of our hypothesis that melatonin is involved in the regulation of *Zugunruhe* could not be confirmed during spring migration.

One interpretation of the results is that melatonin is not causally involved in the expression of *Zugunruhe*. Rather, *Zugunruhe* may depend on unknown factors that control the two correlated variables. For example, melatonin could regulate metabolism and body temperature during a stopover to optimize refuelling, and there is evidence that garden warblers and blackcaps indeed substantially decrease their body temperature during stopover [17,18]. However, one alternative explanation is that melatonin may have a stronger regulatory power on *Zugunruhe* during autumn migration compared to spring migration. This possibility was suggested already by the study of Fusani & Gwinner [14], where we found that melatonin levels of control birds in spring were significantly lower than in autumn, and the effects of the food deprivation protocol on melatonin levels and *Zugunruhe* were much larger in autumn than in spring.

In this work, we tested whether a temporary increase of circulating melatonin induces a reduction of *Zugunruhe* during autumn migration. To accomplish this aim we repeated the melatonin treatment as in [15] on both garden warblers and blackcaps at a stopover site in southern Germany during autumnal migration. Rather surprisingly, although not unexpectedly, we found strong effects of melatonin on *Zugunruhe*, which was reduced in the night following the treatment. Therefore, this work confirms that melatonin participates in the regulation of migratory restlessness in *Sylvia* warblers, and provides novel evidence for seasonal variations in the sensitivity and modulation of the migratory programme.

## Materials and methods

### Experimental design

The experimental design was based on an ‘overnight’ approach: birds were caught in the morning and hosted in registration cages to record locomotory activity until their release in the following morning. During this period, the birds were exposed to two experimental treatments: availability or absence of food, and administration of melatonin or a control treatment. Treatments were crossed in a two-factor ANOVA design: food and melatonin; food and no melatonin; no food and melatonin; no food and no melatonin.

### Study site and experimental procedures

The study was conducted in Radolfzell, in south-western Germany (47.729°N, 8.998°E) close to lake Constance, between August 18^th^ and September 7^th^ 2012. We focused on two target species, blackcaps (*Sylvia atricapilla*) and garden warblers (*Sylvia borin*). Eighty-eight birds - 60 blackcaps (BC) and 28 garden warblers (GW) - were caught with mist-nets before 12:00 h (GMT + 1) at the Mettnau ringing station, which belongs to the Max-Planck-Institut für Ornithologie. A series of morphological and physiological variables were measured following standardized EURING procedures [19], including sex (*S. atricapilla*), age (first year or older than one year), body weight, size of the pectoral muscles on a 0-3 scale, and extent of subcutaneous fat on a 0-8 scale. Birds with fat score <1 were excluded from the experiment to reduce the risks of negative effects of the temporary captivity. By 13:00 h the birds were transported to the laboratory of the Max-Planck-Institut für Ornithologie which is 5 km from the ringing station, and were housed in a room with artificial lighting simulating the local photoperiod. Birds were set in individual, custom-built fabric cages so that they were visually isolated from each other. Each cage was equipped with an infra-red sensor connected with an activity recorder that registered the locomotor activity of the birds at 2-min intervals details in [14]. The birds were randomly allocated to two groups: one group received a full standard diet consisting of 10 meal worms, 20 g of a mixture of dry insect food, banana and boiled egg [14], the other group received no food. Water was available *ad libitum* for both groups. All birds were left undisturbed until one hour before civil twilight when half of them was subjected to the melatonin treatment. The melatonin treated birds were treated with 100 μl Eucerin (*Eucerinum anhydricum*, Bayersdorf AG, Germany) cream containing 39 μg of melatonin. Control birds were treated with 100 μl Eucerin. The cream was applied to the area of naked skin between the dorsal feather ridge, the wings and the neck. This treatment protocol had been previously shown to increase plasma melatonin for at least four hours [15,16]. The treatment was conducted before civil sunset at 19:30 h ± 30 min, after which the birds were left undisturbed until morning. Of the 49 individuals that received food (32 BC and 17 GW), 23 were control treated (15 BC and 8 GW) while 26 were melatonin treated (17 BC and 9 GW). Among the 40 unfed birds (28 BC and 11 GW), 21 were control treated (14 BC and 7 GW) and 19 melatonin treated (14 BC AND 5 GW). To confirm the efficacy of the melatonin treatment, at 24:00 h ± 30 min we collected a 100μl blood sample into heparinized capillaries after puncturing the alar vein with a 26-gauge sterile needle under red light from a total of 21 vs. 19 control treated birds, specifically: food and melatonin, N = 13 (9 BC and 4 GW); no food and melatonin, N = 8 (6 BC and 2 GW); food and no melatonin, N = 12 (7 BC and 5 GW); no food and no melatonin, N = 7 (5 BC and 2 GW). The samples were centrifuged at 3000 rpm for 5 minutes and after collection the plasma was frozen at -20°C until analysis. All birds were released the following morning after 07:00 h. The body mass of birds was measured when birds were set into the cages and before release. All the experimental procedures were approved by the responsible authorities (Regierungspräsidium Freiburg; AZ no. 35-9185.81/G-12/67).

### Melatonin measurement

The plasma concentration of melatonin was determined by direct radioimmunoassay (RIA) using standard techniques already used in previous studies [14,15] details of the methods in [16]. The standard curve and sample concentrations were calculated with Immunofit 3.0 (Beckman Inc. Fullerton, CA), using a four parameter logistic curve fit. The lower detection limit of the standard curve was determined as the first value outside the 95% confidence intervals for the zero standard (Bmax) and was 2.3 pg/ml. The intra-assay coefficient of variation was 1.4% and the intra-extraction coefficient of variation was 2.4%. Melatonin concentrations were adjusted for individual recoveries (mean ± std recoveries 86.0% ± 6.0%).

### Data analysis

The activity recording system measured the number of times the infrared sensor was activated during each 2 min period. From these values, we calculated the average activity for the night period between 20:00- 06:00 h (GMT + 1). The beginning and end of this period was defined according to the local civil twilight table for the period 15^th^of August – 10^th^ of September. We also divided the total night in two sub-periods: 20:00- 24:00 h (night 1) and 24:02- 06:00 h (night 2), but because half of the birds were bled at midnight we only used the activity data from the first period for the statistical analysis.

To analyse whether the melatonin treatment affected the plasma melatonin concentration we ran a two-way ANOVA with melatonin and food treatments as between-subjects factors using the subset of birds that were bled for melatonin. To study the effect of melatonin treatment on *Zugunruhe* we included all birds, but used activity data from the first half of the night, only, because the blood sampling may have affected subsequent activity. We investigated the effects of species, melatonin treatment, food availability, and fat on *Zugunruhe*. Explorative analyses showed that the residuals of the activity data were not normally distributed. Therefore, we used a Generalised Additive Model (GAM), from the ‘mgcv’ R package [20-22]. To optimise the model, we fitted a quasi-Poisson distribution with a log link function. Species, melatonin treatment, and food availability were modelled as factors. Because fat did not contain enough categories (less than 5) to allow the use of a smoother we also modelled fat as a factor with 4 levels, comparing fat classes 2-4 with fat class 1. Data distribution could not be normalized using standard transformation methods, therefore we used a quasi-Poisson distribution [23] with variance proportional to the mean and a log-link function to constrain the estimates to be positive. This quasi-likelihood approach assumes that the scale parameter Φ of the distribution is unknown and can consequently account for more overdispersion than the classical Poisson distribution [20]. In addition we analysed the change in body mass by using a proportional change ([body mass when put into cage – body mass at release]/[body mass when put into cage + body mass at release]) that is independent of absolute change which may be species-specific. We investigated whether the change in body mass was related to melatonin and food treatment and whether the birds were bled or not. In this model we used a GAM with a Gaussian distribution. Residuals of the models were analysed using graphical methods [24] for homogeneity of variance, violation of normality assumptions and departures from the model assumptions or other anomalies in the data and in the model fit. All data were analysed using R version 3.0.1 [25] (http://www.r-project.org/) and significance was set at *P* < 0.05 for all the performed analyses.

## Result*s*

### Effects of the melatonin treatment on plasma melatonin concentrations

**Figure 1:**
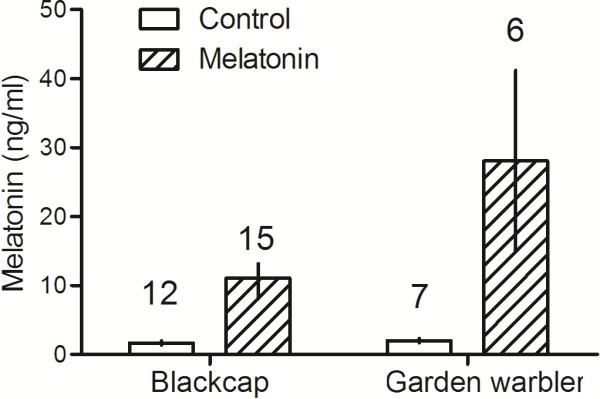
**Mean (± sem) plasma melatonin concentrations in blackcaps (left) and garden warblers (right) at 24:00 h, i.e. 4:30 ± 0:30 hrs after the melatonin treatment.** The treatment significantly increased plasma melatonin concentration in both species (see text for details). Numbers above columns indicate sample sizes.

As expected from our previous studies [15,16], melatonin treatment significantly elevated plasma melatonin concentrations (ANOVA, F_1,40_ = 7.182, *P =* 0.011; Figure 1). Food availability (F_1,40_ = 1.058, *P* = 0.310) and the interaction between melatonin and food availability (F_1,42_ = 0.991, *P* = 0.326) had no significant effect on melatonin levels. We did not include species in the model because we did not expect a different effect of the treatment between species; when included, the factor had no significant effect (F_1,42_ = 1.480, *P* = 0.233).

### Effects of the melatonin treatment on Zugunruhe

**Figure 2:**
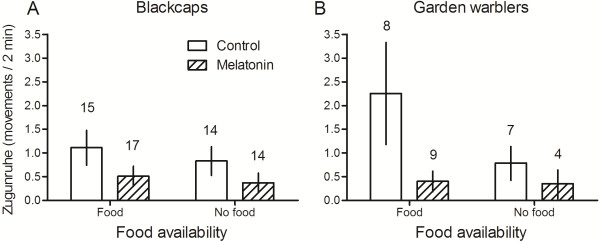
**Mean (± sem) *Zugunruhe *(number of movements during 2-min periods) shown by blackcaps (A) and garden warblers (B) in the night following the melatonin treatment.** There was a significant effect of the melatonin treatment and fat on *Zugunruhe* (see text and Table 1 for details). Numbers above columns indicate sample sizes.

The analyses showed that the melatonin treatment and fat significantly affected *Zugunruhe* in blackcaps and garden warblers (Table 1, Figure 2). First, we ran a model including species, melatonin treatment, food availability, the fat classes 1 to 4, and the interaction between melatonin treatment and food availability. Because the interaction was not significant (t = -0.262, *P* =0.794) it was removed from the final model. Food availability had no significant effect, but there was a trend in that birds receiving food showed more *Zugunruhe* than birds that did not receive food (Table 1). Furthermore, garden warblers tended to show a lower amount of *Zugunruhe* than blackcaps (Table 1; Figure 2). The melatonin treatment significantly decreased *Zugunruhe* compared to the control treatment in both species (Table 1; Figure 2 A, B) and birds with fat classes 2 to 4 showed a significantly higher amount of *Zugunruhe* than birds with fat class 1 (Table 1; Figure 2).

**Table 1:** **Parametric components (estimated parameters, standard errors, *t*-values and *P*-values) of the generalized additive model analysis on factors affecting *Zugunruhe***

	**Estimate**	**Std. error**	**t value**	***P***
(Intercept)	-1.456	0.312	-4.669	<0.001
Species	-0.519	0.281	-1.850	0.068
Melatonin treatment	-0.596	0.253	-2.355	0.021
Food treatment	-0.448	0.257	-1.746	0.085
Fat 1	0			
Fat 2	1.279	0.370	3.459	<0.001
Fat 3	1.455	0.386	3.771	0.006
Fat 4	1.272	0.450	2.827	0.021

The model was fitted by using a quasi-Poisson distribution with log-link function and a Generalized Cross-Validation criterion (GCV) as model optimizer. In the model a fat score of 1 served as the baseline level to which the other fat score levels were compared to.

adjusted R^2^ = 0.168 (expl. deviance = 22.5%), GCV score = 0.478, N = 88.

### Changes in body mass

**Figure 3:**
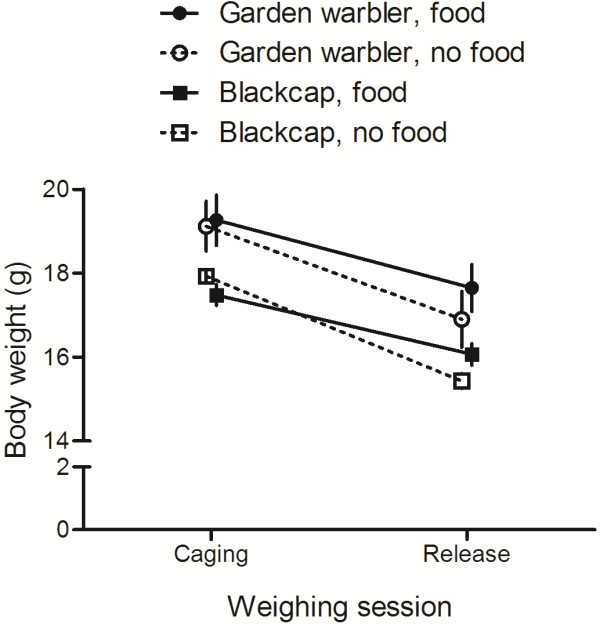
**Mean (± sem) body weight of blackcaps and garden warblers at the time of putting them into the cage and at release.** The availability of food had a significant effect on the variation of body weight between caging and release (see text and Table 2 for details). Numbers above columns indicate sample sizes.

The relative change in body mass between the time when the birds were transferred to the cages and when they were released in the next morning depended on the food treatment (Figure 3 and Table 2). Birds that did not receive food lost significantly more weight than birds that received food (Figure 3). Other factors did not influence the extent of body mass variation (Table 2).

**Table 2:** **Parametric components (estimated parameters, standard errors, *t*-values and *P*-values) of the generalized additive model analysis on factors affecting a change in body mass**

	**Estimate**	**Std. error**	**t value**	***P***
(Intercept)	0.04528	0.00690	6.554	<0.001
Species	-0.00535	0.00697	-0.767	0.445
Melatonin treatment	-0.00784	0.00652	-1.203	0.232
Food treatment	0.02798	0.00659	4.242	<0.001
Blood sampling	0.00767	0.00653	1.175	0.243

The model was fitted by using a Gaussian distribution and a Generalized Cross-Validation criterion (GCV) as model optimizer.

adjusted R^2^ = 0.185 (expl. deviance = 22.3%), GCV score = 0.001, N = 87.

## Discussion

In this study, we tested if administration of melatonin affects the intensity of *Zugunruhe* during autumnal migration in two nocturnal migrants, the garden warbler and the blackcap. We found that an experimental increase in plasma concentrations of melatonin was accompanied by a substantial reduction of *Zugunruhe*. Thus, we demonstrated that melatonin can influence the migratory programme of *Sylvia* warblers during autumnal migration. This hypothesis was suggested by a first series of studies on garden warblers and blackcaps ([4,7], reviewed by [8,9,14]) illustrating that the amplitude of melatonin at night decreases during migratory periods compared to other stages of the annual cycle [7], but increases when migration is temporarily interrupted during a simulated stopover induced by fasting and re-feeding [14,26].

The results of the present study are in contrast with those of a similar experiment performed during spring migration, in which no effect of melatonin on *Zugunruhe* was found [15]. What are the potential reasons for these differences in results between the previous and the current study? Methodological issues can be ruled out, because we used the same cages and recording apparatus - i.e. we transported them to Radolfzell - the same type of food, and the same melatonin treatment protocol in the two studies. What differed between the two studies was the location, the experimental birds, and the migratory season. Whereas we do not see any reason why the results of the test should be affected by the location itself, it is likely that the relative geographical position of the study site along the migratory route may have played a role. Radolfzell is north of the Alps and when passing this continental region on their southward autumn migration, birds have not yet encountered any major ecological barrier and are at the beginning of migration. Thus, they are probably receptive to environmental or endogenous cues that may help them to optimize their migratory strategy. In contrast, Ponza (the site of the spring migration study) is a Mediterranean island that receives mainly migrants from Northern Africa and possibly Sicily and neighbouring islands. In any case, the birds must have crossed at least 500 km of open sea until they finally reached Ponza. Thus, birds caught at the two sites differ in their metabolic state, i.e. the Radolfzell birds are likely to be in an anabolic state as they are building up reserves in food-rich areas before the toughest part of their migration, whereas the birds caught in Ponza have just passed the most difficult segment of their journey (i.e. they have successfully crossed the Sahara and the Mediterranean Sea) and are in a catabolic state, because they may have lost most of their reserves during these crossings. Such physiological differences between birds at the two sites could have been responsible for the differences in the response to the melatonin treatment. Extended fasting and sustained flight periods cause reduction of proteinaceous tissues, and digestive tract and liver are catabolised more than other tissues [27,28]. In order to refuel and substantially gain mass and improve body condition following an extended flight period, birds must rebuild their digestive tract. Efficient rebuilding requires protein rich diet [29], which might be not available on Ponza. Thus, birds might be trying to leave Ponza as soon as possible and resist to any alteration of their migratory strategy. In fact, in spring melatonin did not affect the migratory programme whereas food availability did [15]. Moreover, birds caught in Ponza are in the final segment of their migration, and might be under time constraints. In the future, we will study the availability of food in Ponza and the anabolic/catabolic state of birds to further investigate this aspect.

A second factor that may underlie the differences between the two studies is the migratory phenotype of the studied populations. Garden warblers and blackcaps have vast breeding areas that span from southern Europe to Scandinavia and for blackcaps even include sedentary populations in in the tropics [30]. Whereas garden warblers are long-distance migrants, blackcaps differ considerably in migratory behaviour. High latitude breeding blackcaps are long-distance migrants that winter in sub-Saharan Africa, more southerly populations are short-distance or partial migrants that move only a few hundred kilometres or not at all, and – as mentioned above – tropical Cape Verde blackcaps are sedentary [30,31]. During spring migration we used only long-distance migrating garden warblers [15]. In the current experiment, the sample of blackcaps could have included short-distance migrants, which might have a less rigid migratory programme than long-distance migrants [32] and thus may be more responsive to environmental and endogenous cues. However, because long-distance migratory garden warblers responded similarly to melatonin treatment during autumn migration than blackcaps, we consider this explanation unlikely.

Finally, spring and autumn migration are separate stages of the annual cycle that have been shown to differ in many aspects (reviewed by [33,34]). For example, birds migrate faster during spring than during autumn [35-38], most likely because of fitness advantages of early arrival in the breeding grounds when competing for high-quality territories and mates [39]. Spring and autumn migration also differ in physiological aspects including hormonal control mechanisms. During spring migration long-distance migrants start to develop their gonads and the release of testosterone is increasing along the migratory route [40]. In female and male white-crowned sparrows (*Zonotricichia leucophrys*) testosterone has been demonstrated to be involved in pre-migratory fattening and the expression of *Zugunruhe* during spring migration (reviewed by [34,41,42]), but autumn migration is unaffected by testosterone [33]. Thus, it is possible that sex steroids such as testosterone influence the motivation to continue migration during spring, thus suppressing any antagonistic effect that melatonin may have on the migratory drive. Several brain regions have both androgen/oestrogen receptors and melatonin receptors [43,44], so an interaction between the two control systems at the central level is possible. In autumn, when testosterone does not play a role in the control of migratory activity, melatonin can exert its suppressive effects. Such a differential regulatory influence of melatonin on migratory activity during spring and autumn would be consistent with the data from our earlier study in which food deprivation had a stronger effect on both *Zugunruhe* and melatonin concentrations in autumn than in spring [14].

What could be the mechanism through which melatonin affects *Zugunruhe*? Melatonin may have direct and indirect effects. Most of the avian brain is sensitive to melatonin, as shown by the widespread distribution of melatonin receptors in almost all brain regions of several birds species [44-46] including *Sylvia* warblers (Fusani & Gahr, unpublished). Thus, it is conceivable the melatonin acts directly on some area to suppress (or reduce) *Zugunruhe* by influencing circadian oscillators [5,47]. In addition or alternatively, melatonin could act on other pathways that influence locomotor activity. For example, melatonin is a key regulator of body temperature [48] and in birds administration of melatonin reduces body temperature [49]. There is evidence that body temperature is reduced in migratory blackcaps and garden warblers that are resting at night as compared to birds that show *Zugunruhe*[17,18] although such hypothermic responses might be limited to birds in very poor condition [50]. Because a reduction of body temperature may be associated with a decrease of the basal metabolic rate [51], it is likely that melatonin participates in the co-ordination of physiological adaptations required from the transitions between resting and active phases.

It is suggestive that several species of wild plants carry berries in autumn which contain elevated levels of melatonin [52]. In line with this observation, interrupting migration upon consumption of melatonin contained in wild berries would be adaptive in that it would stimulate the permanence in stopover areas with abundant sources of carbohydrates and antioxidants. This interpretation would also explain why birds do not respond to melatonin in spring, when such natural sources of the hormone are not available.

In conclusion, our study indicates that melatonin may be involved in the decision-making of *Sylvia* warblers to either continue autumn migration or to remain at a stop-over site. In combination with the results of our previous study, in which we found no effect of melatonin during spring migration [15], the present results hint at possible differences in the hormonal regulation of spring and autumn migration.

## Competing interests

The authors declare that they have no competing interests.

## Authors’ contributions

LF conceived the study and designed the experiment, conducted the experiment, and wrote the manuscript. WG conceived the study and designed the experiment, performed the statistical analysis, and wrote the manuscript. FC conducted the experiments, contributed to hormone and statistical analyses and to the writing of the manuscript. ARM conducted the experiments and contributed to data elaboration. All authors read and approved the final manuscript.
